# Why Inhibition of IL-23 Lacked Efficacy in Ankylosing Spondylitis

**DOI:** 10.3389/fimmu.2021.614255

**Published:** 2021-03-19

**Authors:** Dennis McGonagle, Abdulla Watad, Kassem Sharif, Charlie Bridgewood

**Affiliations:** ^1^Leeds Institute of Rheumatic and Musculoskeletal Medicine, University of Leeds, Leeds, United Kingdom; ^2^National Institute for Health Research (NIHR), Leeds Biomedical Research Centre (BRC), Leeds Teaching Hospitals, Leeds, United Kingdom; ^3^Department of Medicine ‘B’, Zabludowicz Center for Autoimmune Diseases, Sheba Medical Center, Tel-Hashomer, Israel; ^4^Sackler Faculty of Medicine, Tel-Aviv University, Tel-Aviv, Israel

**Keywords:** IL-23, psoriatic arthritis, ankylosing spondylitis, enthesis, IL-17

## Abstract

The term spondyloarthritis pertains to both axial and peripheral arthritis including ankylosing spondylitis (AS) and psoriatic arthritis (PsA), which is strongly linked to psoriasis and also the arthritis associated with inflammatory bowel disease. The argument supporting the role for IL-23 across the spectrum of SpA comes from 4 sources. First, genome wide associated studies (GWAS) have shown that all the aforementioned disorders exhibit IL-23R pathway SNPs, whereas HLA-B27 is not linked to all of these diseases-hence the IL-23 pathway represents the common genetic denominator. Secondly, experimental animal models have demonstrated a pivotal role for the IL-23/IL-17 axis in SpA related arthropathy that initially manifests as enthesitis, but also synovitis and axial inflammation and also associated aortic root and cutaneous inflammation. Thirdly, the emergent immunology of the human enthesis also supports the presence of IL-23 producing myeloid cells, not just at the enthesis but in other SpA associated sites including skin and gut. Finally, drugs that target the IL-23 pathway show excellent efficacy for skin disease, efficacy for IBD and also in peripheral arthropathy associated with SpA. The apparent failure of IL-23 blockade in the AS which is effectively a spinal polyenthesitis but evidence for efficacy of IL-23 inhibition for peripheral enthesitis in PsA and preliminary suggestions for benefit in axial PsA, raises many questions. Key amongst these is whether spinal inflammation may exhibit entheseal IL-17A production independent of IL-23 but peripheral enthesitis is largely dependent on IL-23 driven IL-17 production. Furthermore, IL-23 blocking strategies in animal models may prevent experimental SpA evolution but not prevent established disease, perhaps pointing towards a role for IL-23 in innate immune disease initiation whereas persistent disease is dependent on memory T-cell responses that drive IL-17A production independently of IL-23, but this needs further study. Furthermore, IL-12/23 posology in inflammatory bowel disease is substantially higher than that used in AS trials which merits consideration. Therefore, the IL-23 pathway is centrally involved in the SpA concept but the nuances and intricacies in axial inflammation that suggest non-response to IL-23 antagonism await formal definition. The absence of comparative immunology between the different skeletal sites renders explanations purely hypothetical at this juncture.

## Introduction

The seminal clinical observations by Moll and Wright in the 1970s classified several diseases under the umbrella term of Spondyloarthorpathy (SpA) based on shared clinical and immunological features (1). These conditions included ankylosing spondylitis (AS), psoriatic arthritis (PsA) (and by extension of the psoriasis spectrum), inflammatory bowel disease (IBD) associated arthropathy including Crohn’s disease and ulcerative colitis, enterogenic and urethrogenic reactive arthritis and anterior uveitis which is also associated with these conditions (2, 3). The common theme across these disorders was axial inflammation, peripheral lower limb oligoarthritis, enthesitis in some cases, a link to infection or intestinal dysfunction and negativity for rheumatoid factor (4, 5). A unified pathological understanding for the SpA associated arthropathy was not proposed in the original iteration of the concept.

Following on shortly after the Moll and Wright’s SpA concept was the discovery of HLA-B27 that was associated with AS, PsA axial disease, IBD related axial arthritis, anterior uveitis and reactive arthritis (6–8). However, IBD itself or IBD related peripheral arthropathy was not associated with HLA-B27. The clinical features of Bechet’s disease (BD) resulted in the investigators also proposing this to represent a member of the SpA concept in a paper that has been cited highly over four decades (9). The absence of sacroiliitis and the lack of a strong association with HLA-B27 meant that BD was never widely adopted in this proposed classification scheme. However, alluded to in the following discussion, GWAS studies have shown that the IL-23 pathway related genetic polymorphisms occur along the entire SpA arthropathy spectrum including ankylosing spondylitis and psoriatic arthritis, in psoriasis and inflammatory bowel disease and indeed in BD, thus completely vindicating the entire concept alluded to by Moll and Wright (9, 10).

## Current Therapy in AS

The current therapy options in AS include an anti-TNF agents for subjects that fail to respond to NSAIDS (Non-steroidal anti-inflammatory drugs). If anti-TNF is contraindicated or if there is loss of efficacy to anti-TNFs, one of two anti-IL-17A blockers can be considered with the provision that these agents should not be used for active associated IBD (11). The JAK inhibitors are likely to enter the clinical arena in AS in the coming years (12). Although guselkumab and ustekinumab may have some efficacy in PsA related axial disease (13, 14) there is no evidence for efficacy of this class of agent in AS from trials with ustekinumab and other p19 blocker risankizumab (15, 16).

## IL-23/IL-17 Axis

When naïve T-cells encounter a cognate antigen in lymphoid tissues, they have the ability to differentiate into effector T-cells, depending on the local microenvironment. This involves MHC peptide presentation to the T-cell receptor (TCR) (signal 1) and then co-activation with CD80/86 binding to CD28 (signal 2) on T-cells (17). In humans, cytokine stimuli such as IL‐1β, IL‐6, IL‐21, and/or IL‐23 can drive IL‐17 production from T-cells, with the best described of these being CD4+ Th17 cells and CD8+ Tc17 cells (18). These IL-23 activated T-cells also secrete a range of other cytokines such as IL-17F, IL-22 and TNF (18).

## The IL-23 Pathway Genetic Argument in SpA Spectrum Disorders

**Figure 1:**
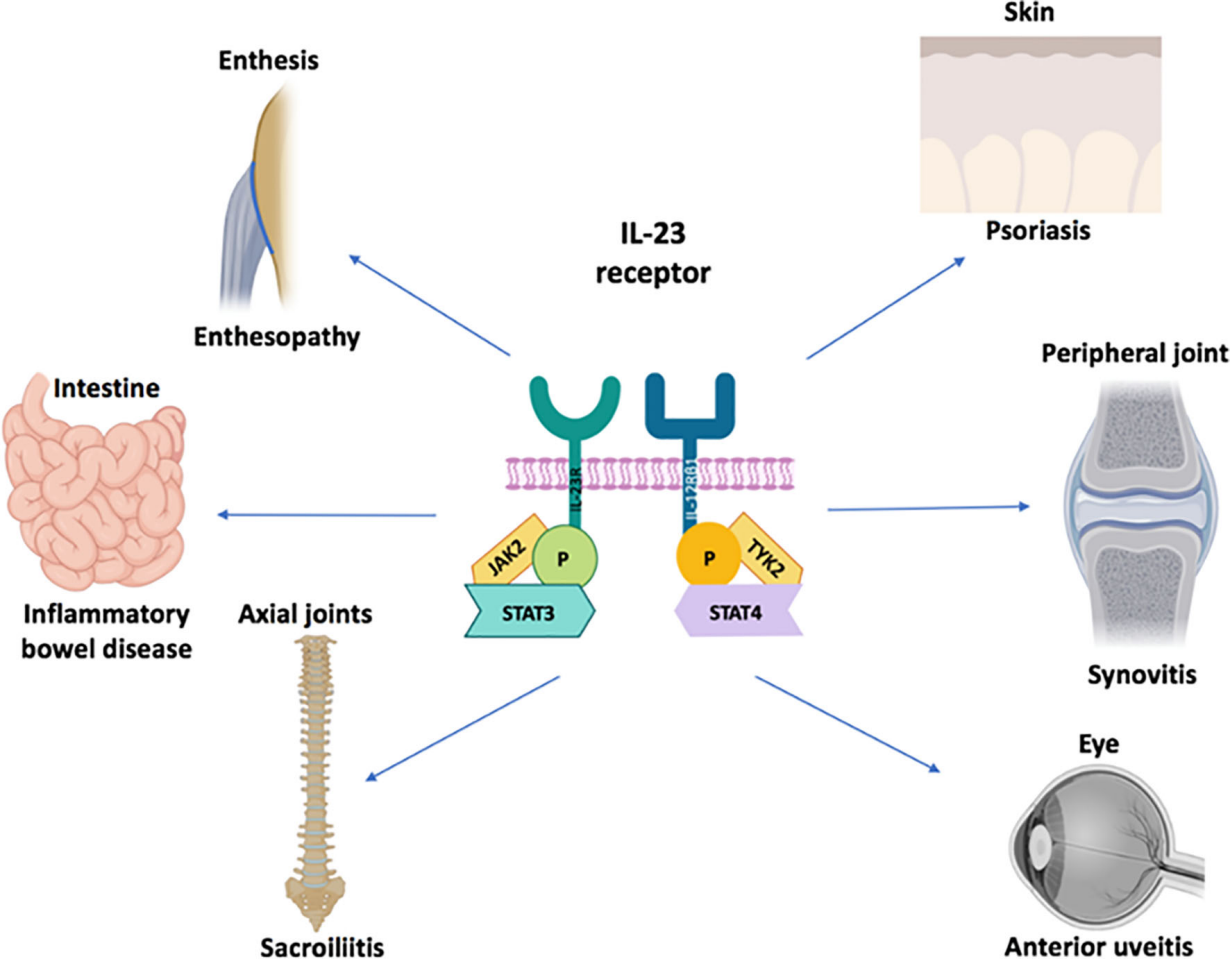
IL-23 receptor polymorphism associated disorders. The IL-23 cytokine pathway has been firmly linked to ocular, cutaneous, intestinal disease and arthropathy at the genetic and cellular immunology level. There is considerable immunological heterogeneity in different organ responses. For example, the TNF-Fc fusion protein, etanercept does not work for IBD and is generally not effective for anterior uveitis. The role of IL-23 blockers for anterior uveitis awaits further definition but it is generally considered that anti-IL-17A blockers are not effective in this SpA domain All the agents of that antagonise the IL-23/17 axis show remarkable efficacy for psoriasis compared to joint disease. With respect to gut disease, the IL-17 blockers have an important role in barrier function at this site which may explain exacerbations of IBD under anti-IL-17A therapy. All three cytokines including TNF, IL-17A and IL-23 may be equally important for peripheral arthritis in PsA and also for enthesitis in the peripheral skeleton because good responses are seen following therapeutic antagonism. The anti-p40 IL-12/23 may be less effective than the other three categories of drugs for Psoriatic arthritis although further studies are needed. Finally, only TNF and IL-17 blockers have shown efficacy in the axial skeleton where IL-23 blockade with either p40 or p19 blockers has not worked. The site-specific compartmentalisation of immunity has come into sharp focus in the past few years and likely reflects tissue specific factors and microbiota interactive factors shaping diverse immune responses.

**Figure 2:**
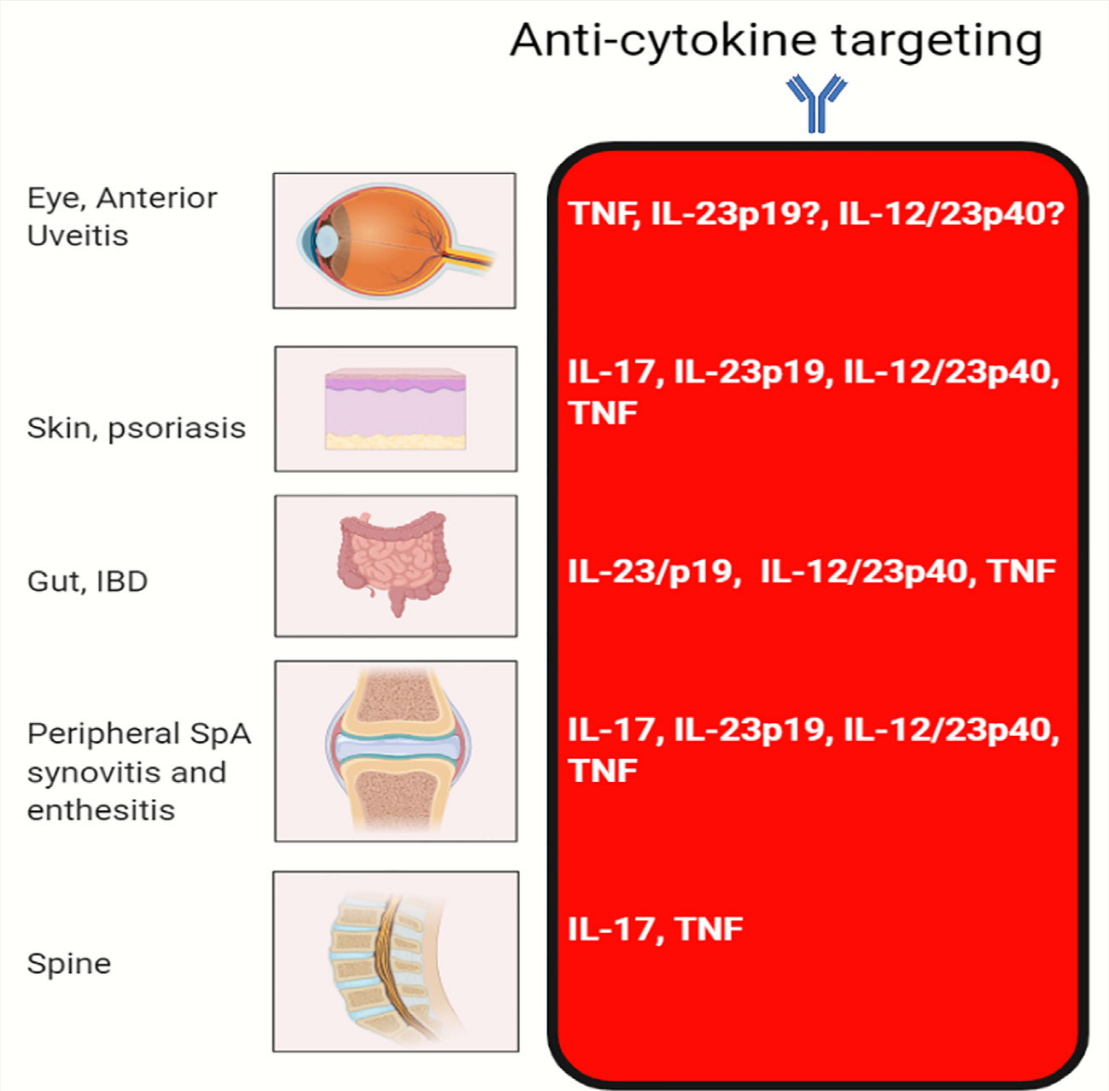
Efficacy of cytokine blocking in different organs. The classical MHC class II associated autoimmune diseases that are characterised by autoantibody production segregate in families and individuals and show a female preponderance. The SpA group of diseases do not show substantial sex differences, have MHC class 1 genetic associations, a lack of specific confirmed disease associated autoantibodies and disease localisation to sites of injury or physical stressing. It is into this mix that genetics and experimental immunology have firmly confirmed the key role for the IL-23 pathway. Given that IL-23 regulates both IL-22 and IL-17, we believe that in addition to immunity including anti-fungal immunity *via* IL-17 regulation that the IL-23 pathway fine tunes tissue repair at sites of injury and physical or chemical stressing as for example in the intestine. TNF-Fc fusion protein, etanercept does not work for IBD and generally not effective for anterior uveitis.

Remarkably, IL-23R polymorphisms have been reported across all of the aforementioned categories of disease but not in classical autoimmunity (**Figure 1**). Furthermore, several SNPs related to the IL-23 pathway including those in the IL-23 cytokine itself, downstream JAK2 and Tyk2, STAT3 and IL-17RA signalling have also been reported across all of these diseases (5, 9, 19). A wealth of other genetic polymorphism data has strongly vindicated these findings insofar that classical autoimmune diseases have a completely different non-IL-23 pathway related genetic architecture (20). The IL-23R pathway SNPs are also associated with IBD (21) and BD (22), thus reinvigorating the historical ties with SpA as suggested by Moll and Wright and colleagues. The SNP in the IL-23R (R381Q) confers protection from IBD, AS and psoriasis (23–25). At a functional level, it results in a loss of function and less STAT3 activation and thus less induced IL-17 from T-cells (26, 27). Thus, it appears that “completely normal” IL-23 pathway signalling and functioning, which is comparatively higher than in subjects with the R381Q polymorphism is linked to AS. It might be theorised that anti-IL-23 therapy would reduce this further and align it with production levels associated with the protective allele. However, this has not been corroborated from trials in AS. While IL-23 pathway is genetically implicated in all the aforementioned tissues, the difference in relative contribution of IL-23 and other cytokines to the different SpA associated diseases shows differential efficacy as demonstrated by clinical trials (**Figure 2**).

## Tissue Microanatomy of IL-23 Pathway and Animal Models

It is well established that the synovium is the primary target of inflammation in RA with autoimmunity directed against citrullinated synovial proteins driving an inflammatory reaction culminating in periarticular joint destruction and erosion, with the well-recognised polyarticular joint destruction phenotype. In the mid-1990s, MRI studies showed that enthesitis was evident in both swollen small and large joints in PsA and SpA in general (28). This resulted in the enthesitis based model for SpA whereby it was proposed cytokine mediated primary inflammatory reactions at the enthesis triggered an adjacent synovitis, tenosynovitis and osteitis (29–31). It was subsequently shown in animal models that dysregulated TNF production at the enthesis triggered polyarticular joint destruction, which further validated enthesitis as a mechanism of disease (32, 33).

A seminal paper by Sherlock et al. demonstrated that the normal murine enthesis harboured an IL-23R expressing cell population (34). This model was later confirmed to be Tyk2 dependent (Tyk2 mediates IL-23 signalling) (35) while the same paper found Tyk2 SNPs correlate with human AS disease progression. In the IL-23 minicricle model, the distal over-expression of the IL-23 cytokine in the murine liver using DNA minicircle technology resulted in a rapid onset of peripheral enthesitis that subsequently spread to the synovium and bone leading to polyarticular joint destruction. In the Sherlock model of IL-23 dependent enthesitis (34), it was subsequently shown by Reinhardt et al. that the majority of IL-17A producing cells in the normal murine enthesis were IL-23R expressing γδ T-cells (36). This population of cells are heterogeneous and carry out diverse functions including early innate immune responses, priming of adaptive immunity as well as prominent roles in tissue repair (37). The role of IL-23 in the SpA concept was strongly cemented in this model by the induction of psoriasiform skin inflammation, aortic root inflammation and also the development of axial inflammation (34). Other investigators using the same minicircle technology emphasised the role of severe synovitis and bone erosion and rheumatoid arthritis like features (38).

Of course, there are several independent animal models showing the pivotal role of IL-23 in experimental gut inflammation and reactive type arthritis (39, 40). A body of emergent research has also linked intestinal microbiota to the IL-23/17 axis interdependence and cross-regulation with this being an area of active research (41–44). Another example of an IL-23 dependent SpA comes from the SKG mouse model that exhibits many of the SpA features in an IL-23/17 axis dependent pathway (45). The SKG contains a point mutation in the ZAP-70 gene, yielding reduced T-cell receptor signalling and following administration with fungal or bacterial adjuvants develops multi-organ inflammation and a SpA like disease (45). Collectively, these models support the idea that inflammation that is topographically enthesis centred drives disease (31).

## Emerging Immunology of the IL-23 Pathway and IL-23/17 Axis in Human SpA

Clinical trials in man dissect human disease immunopathogenesis and it is important to turn to these, in order to better understand human enthesitis. First, IL-17A blockers have proven efficacy for both peripheral and axial SpA including evidence for efficacy for isolated enthesitis as a secondary outcome measure (46–50). Likewise, the published literature shows efficacy for IL-12/IL-23 p40 blockers for peripheral PsA and for isolated enthesitis (30, 51–53). Recent studies have also shown that IL-23p19 blockade is effective for peripheral synovitis and related enthesitis (54, 55). These findings alone point towards a biological role for IL-23 at the non-axial peripheral enthesis (56), but what is the biological basis for this?

Following on from the Sherlock et al. study (34), our group investigated the presence of IL-23/17 axis cytokines at the normal human spinal enthesis. We defined normal spinal enthesis bone and soft tissue resident IL-23R expressing group 3 innate lymphoid cells (57). IL-23/IL-1β stimulation of normal human enthesis tissue resulted in upregulation of IL-17A and IL-17F transcript (57). Moreover, in humans we previously reported the presence of macrophages in acute enthesitis (58). This raised the possibility that local IL-23 production may be possible at the human enthesis and it was subsequently shown that the normal enthesis contains IL-23 inducible protein production from CD14+ myeloid cell following bacterial or fungal stimulation (59). We also found that this IL-23 secretion could be attenuated by the addition of PDE4 blockers which may be relevant translationally since antagonism of this pathway shows efficacy for peripheral enthesitis in PsA in man (56). Both TNF and IL-17A are able to also induce osteogenesis *in vitro* in MSC from the spinal enthesis (60, 61).

## Complexity of the IL-23 Pathway in the Spine and Other SpA Features

Since the failure of IL-23 blocking in the AS, there has been great scientific speculation into the reason why (62). Remarkably, although the SpA group of conditions are closely interlinked, they also exhibit a differential immunopathology between different sites that is best encapsulated in the non-efficacy of therapies in some domains (**Table 1**). For example, the TNF fusion protein etanercept shows efficacy for the skeleton but not in the gut (68). Likewise, IL-17A blockers show impressive efficacy in the skin and good efficacy in the skeleton but are ineffective in the gut and in some circumstances are associated with IBD exacerbation (69). Laboratory research following the failed human trials of anti-IL-17A in Crohn’s disease led to observations that γδ T-cell IL-17A production in the gut is produced independent of IL-23R signalling where IL -17 signalling was required for maintaining intestinal occludin junctions (70).

**Table 1:** Spondyloarthritis spectrum disease heterogeneity in immunotherapy responses.

Pathway	Agent	Adverse Event	Immunopathology	Recommendations	References
TNF	Infliximab, Etanercept, Adalimumab, Certolizumab pegol, Golimumab	Peripheral arthralgia in IBD therapy, Paradoxical psoriasis	Paradoxical upregulation of interferon pathways	Switch to IL-23 or IL-17 (except in IBD) inhibitors	(63, 64)
TNF	(Etanercept)	Uveitis, lack of efficacy in IBD	Mechanism unclear but in gut might be linked to fact antibodies may be linked to antibody dependent cytotoxicity for myeloid cells	Switch to a different TNF blocker	(63, 64)
IL-17	Secukinumab, Brodalumab, Ixekizumab	Inflammatory bowel disease	Dysregulation of the intestinal epithelial permeability which is regulated by IL-17A (tight junction).	Switch to TNF or IL-23 inhibitors	(65)
IL-23 (p40 and p19 blockers)	Ustekinumab, Rasinkizumab	Lack of evidence for efficacy in ankylosing spondylitis	Not understood but likely IL-17A production independently of IL-23		(15, 16)
α4β7 integrin	Vedolizumab	Sacroiliitis and synovitis	Abnormal intestinal barrier function and access of bacterial antigens, cytokines, adjuvants and pathogen-associated molecular pattern molecules to the systemic circulation and deposition in the peripheral skeleton at regions of entheseal tissue.	Switch to TNF or IL-12/23 blockers	(66, 67)

Given the aforementioned efficacy of IL-17 blockade in axial disease and the non-efficacy of IL-17A inhibition in the gut, the question arises as to whether there may be pathway for IL-17 production in the spine that is independent of IL-23 that may account for the curious reported lack of efficacy for IL-23 pathway inhibition in axial disease. Two trials of IL-23 pathway blockade including p40 and p19 blockade failed to show efficacy in AS, although marginal non-statistically significant improvements in CRP and subtle MRI improvements were evident under p40 antagonism (15, 16). There are two phase II trials of p19 blockers showing efficacy in psoriatic arthritis peripheral arthropathy including peripheral enthesitis (54, 55, 71). This has thrown up a new conundrum- how can a drug work for peripheral skeletal enthesitis but not axial enthesitis that underpins most of the AS pathology outside the sacroiliac joint. One important difference may be the presence of synovio-entheseal complexes in the peripheral skeleton but not in the spine (72).

## Emergent Cellular Players in the Non-Linearity Between IL-23 and IL-17 Pathways in SpA

Human γδ T-cells are classified into two major groups- δ1 and δ2 (73). We have explored the concept that there may be heterogeneity in these populations in man. Both the normal spinal entheseal soft tissue and peri-entheseal bone have resident γδ T-cell populations with these being more numerous in the peri-entheseal bone (74). In the enthesis resident γδ T-cell populations, we found that the δ1 population lacked IL-23R expression but that the δ2 population expressed this receptor. Only the δ2 population upregulated IL-17A in response to IL-23 signalling. However, both populations could be induced to express IL-17A upon PMA or anti-CD3/CD28 stimulation (74). Hence, the complexity of the IL-23 pathway extends to the spine and our results indicate that IL-17A, a key cytokine in AS and spinal inflammation, may not depend exclusively on IL-23. Accordingly, the IL-23/IL-17A axis is a two-sided coin with IL-17A production independent of IL-23 having very different biological consequences for gut and skeletal inflammation with IL-17A blockade in the former being detrimental but potentially beneficial in the latter (75, 76). In recent times, other theories have emerged of IL-17 secretion independent of IL-23. Mucosal-associated invariant T (MAIT) cells, are specialised innate-like T-cells that serve to bridge innate and adaptive immunity. MAITs are activated by conserved bacterial ligands which are derived from vitamin B biosynthesis, which are presented by the MHC-class I like MR1 to the TCR (77). Following TCR activation and also stimulation with IL-12 and IL-18, MAIT cells have been shown to secrete IL-17 that is independent of IL-23 (78). The human enthesis also contains conventional T-cells, both CD4+ and CD8+. Both entheseal CD4+ and CD8+ are able to secrete IL-17A following TCR stimulation (anti-CD3/CD28) without the need for additional IL-23 stimulation (79).

## IL-23 Blockade for the Prevention of SpA

The failed phase II trial of risankizumab in AS and the failed phase III ustekinumab trial in AS are responsible for these emergent immune insights (80, 81). This has been explored in the experimental SpA model induced in HLA-B27/Huβ2m transgenic rats that spontaneously develop SpA (82). These animals were either treated prophylactically with anti-IL-23R prior to disease onset or with control injections. Conversely the disease was allowed to fully manifest and then the animals were treated with anti-IL23R antibody or control. These experiments showed that IL-23 blockade was capable of preventing disease evolution but incapable of suppressing arthritis when fully established (82). How, exactly this relates to humans is unclear as the nuances of this rat model and its applicability to human SpA are not completely defined (4). For example, the findings might suggest a key role for memory T-cells that could produce IL-17A independent of IL-23 signalling. However, a role for CD8+ T-cells in HLA-B27 experimental SpA has never been substantiated (83), whereas the genetics of human SpA including HLA-B27, ERAP-1 and several other SNPs tends to incriminate this pathway (4).

There is some preliminary evidence supporting these animal models in humans. It has been recently shown that blocking of the IL-23 pathway with ustekinumab in psoriasis results in the regression of subclinical peripheral enthesopathy (84). Whether IL-23 blocker utilisation in psoriasis subjects will prevent axial inflammation evolution is an interesting and open question. It is worth pointing out that a secondary analysis of the pivotal phase III ustekinumab studies in PsA, showed efficacy in axial PsA including improvement in spinal domain pain (13).

Recent studies in abstract form have shown that patients with PsA enlisted in trials for polysynovitis, but also where 20% of patients had radiographic sacroilitis and back pain, that p19 blockade with guselkumab was associated with improvements in axial symptoms (14). These trials point to the possibility of inflammatory spinal disease immunological heterogeneity with some cases of PsA axial inflammation exhibiting a direct role for IL-23, which is stronger and different from that seen in AS.

## Some Loose Ends With Respect to IL-23 in the Spine

It is unlikely that p19 blockade is interfering with the function of the poorly characterised cytokine IL-39, that also shares the p19 subunit (p19+EBI3) (85). Indeed, this cytokine remains a theoretical cytokine in humans with no evidence for either its formation or its function *in vivo* (85, 86). Hence, at this time it seems that the sole role of p19 blockade in main is on IL-23 and not another as yet ill-characterised cytokine, but further work is needed.

Most of the spinal inflammation in AS occurs in the peri-entheseal bone where disease localisation to this site is related to the HLA-B27 genetic status (58). Our work in human spinal entheses shows a much higher production or induction of IL-23 from the bone side of the enthesis (59). Whether this translates into therapeutics remains an open question and maybe higher doses of p19 blockers are needed to alleviate axial inflammation?

The failed trial of ustekinumab in AS used the 45mg and 90mg dosing regimen but the higher dose was associated with a non-significant CRP reduction and minor improvements in MRI lesions (81). The dose of ustekinumab used in Crohn’s disease includes and 6 mg/kg intravenous loading dose (87) which is potentially the equivalent of 18 months of ustekinumab at the 90mg sc regimen for AS in the failed study. Clearly there is room for dose escalation to formally evaluate these questions. Also, it has been suggested that p40 blockers may restrain the immunoregulatory effects of IL-12 in the skin (88) and likewise there is uncertainty about any negative impact that p40 blockers could be exerting outside of the IL-23 pathway. However, the negative p19 study in AS argues against this.

It must be clearly articulated that translational therapy in man, and not laboratory experimental science is leading the understanding of these pathways. It is noteworthy that p40 blockers were associated with efficacy for axial symptoms in PsA, but it must be acknowledged that HLA-B27 negative axial PsA might represent a different disease from AS (89). A clinical short cut to understanding the dosing issues around IL-23 blockers may come from an evaluation of Crohn’s disease therapy dosing on subjects with concomitant axial disease. Unfortunately, there is no comparative immunology between the spinal and peripheral entheses at this time so this is still largely conjectural.

## Conclusions

For the purposes of this article the term SpA was taken to include the protean manifestations associated with axial inflammation including skin and gut involvement where it has clearly been shown that IL-23 SNPs are a common denominator across the different conditions. It is also clearly evident in experimental and human systems that the IL-23/IL-17 axis is involved in skin, gut and entheseal biology (90). A differential immunopathology exists within these disease domains reflecting the context dependent biology of different tissues that is currently best understood in terms of the barrier function role of IL-17A in the gut. The biological basis for IL-17 production in the spine that is seemingly independent of IL-23 needs verification, and if confirmed raises a vital question as to why IL-17A is so crucial to spinal immunobiology.

This non-linearity between IL-23 and IL-17 also appears to exist in the human spine but this knowledge is presently very rudimentary. Nevertheless, there is preliminary evidence suggesting that the downstream IL-17A pathway in axial biology is regulated in both IL-23 and IL-23 independent pathways. Further work is needed in man including IL-23 posology and careful assessment of disease subtypes and objective measures of inflammation including CRP and MRI appearances. The emergent biology of the IL-23/17 axis in the human skeleton strongly suggests that hidden within the current complexity is an IL-23 pathway, there may be a SpA subgroup with axial inflammation that might still be exploitable therapeutically with antagonism of this pathway.

## Author Contributions

CB, AW, KS and DM all contributed to scientific discussion, writing and figure making for the paper. All authors contributed to the article and approved the submitted version.

## Conflict of Interest

The authors declare that the research was conducted in the absence of any commercial or financial relationships that could be construed as a potential conflict of interest.
